# In Vitro Testing and Clinical Handling of a Novel Implant Positioning Technology for Proximal Humeral Plating

**DOI:** 10.3390/medicina59030450

**Published:** 2023-02-23

**Authors:** Markus Windolf, Dominik Knierzinger, Stefaan Nijs, An Sermon, Michael Blauth, Robert Geoff Richards, Jan Buschbaum

**Affiliations:** 1 AO Research Institute Davos, 7270 Davos, Switzerland; 2 Department of Orthopaedics and Traumatology, Medical University of Innsbruck, 6020 Innsbruck, Austria; 3 Department of Development and Regeneration, University Hospital Leuven, 3000 Leuven, Belgium; 4 University Medical Center Utrecht, 3584 CX Utrecht, The Netherlands; 5 Department of Traumatology, University Hospitals Gasthuisberg, 3000 Leuven, Belgium

**Keywords:** proximal humerus, fracture, plating, surgical navigation, implant positioning, screw length

## Abstract

Background and Objectives: Fractures of the proximal humerus are common, particularly in elderly populations. Anatomical locking plates target stabilization with a multitude of screws spanning into the humeral head. Sound implant placement and screw length determination are key for a successful clinical outcome but are difficult to obtain from planar X-rays. A novel implant positioning technology for proximal humerus plating (Xin1) outputs screw lengths suggestions and plate position based on hole projections in conventional X-ray images. This study investigated the performance of a prototype Xin1 system in a postmortem (in vitro) experiment as well as in a clinical handling test. Materials and Methods: For in vitro testing, twelve shoulders from six anatomical specimens were randomized into two groups to compare the Xin1 technique to the conventional operation in terms of surgical precision, procedure time and X-ray exposure. For the clinical trial, 11 patients undergoing plating of the proximal humerus were included. The aim was to investigate clinical handling of the Xin1 marker clip and to retrospectively evaluate the system performance in a real-life fracture situation. Image pairs before and after insertion of the proximal screws were retrospectively processed to investigate the influence of potential bone fragment shifts on the system output. Results: In the postmortem experiment, the use of the system significantly improved the surgical precision (52% error reduction), procedure time (38% shorter) and radiation exposure (64% less X-rays). Clinical handling demonstrated seamless embedding of the marker clip into existing clinical workflows without adverse events reported. Retrospective X-ray analysis on six eligible patients revealed differences in the calculated screw lengths of ≤2 mm before and after screw insertion for five patients. In one patient, the screw lengths differed up to 8 mm, which might indicate displacement of the head fragment. Conclusions: Results suggest a strong potential of the Xin1 assistance technology to enhance the surgical procedure and patient outcomes in the rising incidence of osteoporotic humeral fractures. Robust performance in a real-life fracture situation was observed. In-depth validation of the system is, however, needed before placing it into clinical practice.

## 1. Introduction

Proximal humerus fractures are among the most common injuries [1,2]. Plate osteosynthesis is a well-established and frequently employed treatment option in which the fracture is stabilized by an angular stable plate and four-to-nine locking screws inserted into the humeral head segment. The adequate selection of screw lengths is of utmost importance. Too-short screws may not anchor in the subchondral bone, resulting in compromised support and increased failure risk of fixation, particularly in osteoporotic patients [3]. Longer screws can reduce the risk of mechanical complication; too long screws, though, penetrate through the humeral head into the joint, with severe consequences such as revision surgery.

Adequate plate positioning [4] and determining correct screw lengths [3] are particularly demanding tasks. Intraoperatively judging the three-dimensional (3D) spherical anatomy of the humeral head in combination with inclined screw trajectories from planar fluoroscopic images render this process difficult, resulting in prolonged operational time and increased X-ray exposure.

We recently proposed a simple and generic tracking technology (Xin1—Implant Positioning Assistance) utilizing conventional radiographic images to determine 3D positions and orientations of implants, instruments and anatomy [5]. The system utilizes lens-shaped hole projections in the X-ray images to compute spatial orientations. Among other applications, the principle was also developed into a module for humeral plating [5]. A metal clip with holes is attached to the implant and serves as a reference. Based on two X-ray images, a software algorithm estimates the humeral head position and calculates the required screw lengths for proximal humerus plating. 

The purpose of this study was (1) to assess the performance of the Xin1 system for application at the humerus in comparison to the manual procedure in terms of accuracy, X-ray exposure and procedure time in a postmortem study; and (2) to investigate performance and handling of the system in a clinical fracture situation by retrospectivly comparing the system output before and after screw insertion as a measure for the influence of potential bone fragment shifts.

## 2. Materials and Methods

### 2.1. Xin1 System

The Xin1 technology is a generic, X-ray-based tracking and navigation concept, designed to assist implant positioning in various orthopedic applications. The underlying principle is based on feature extraction from projections of cylindrical holes in X-ray images for determining the spatial alignment of the implant and anatomy. It was first described by Windolf et al. [6] for the distal interlocking of intramedullary nails and later detailed and abstracted in [5]. The system requires a radiopaque reference marker with holes, a conventional C-arm and a computing unit with proprietary image processing software. 

**Figure 1:**
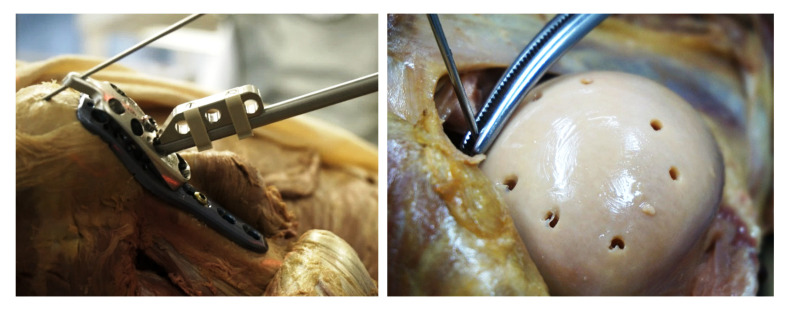
Postmortem experiment. (**Left**): Reference marker clip attached to a conventional drill sleeve and positioned in the center hole of the PHILOS plate (screw 7, D-level) with guide block. Three Xin1 tracking holes arranged in a defined pattern are integrated in the marker body. (**Right**): Over-drilled pilot holes after dislocation of the humeral head. A K-wire was inserted until touching the screw tip to measure the tip-to-joint distance (TJD).

**Figure 2:**
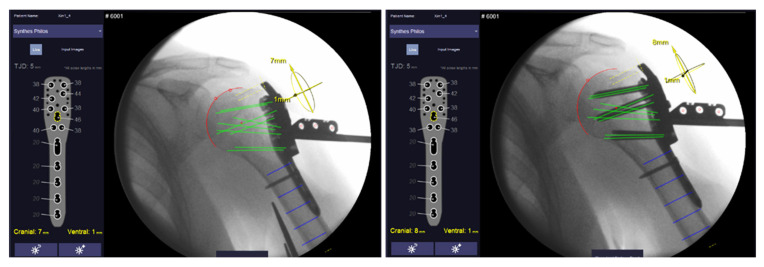
User interface of the Xin1 software for proximal humeral plating showing the semi-automatically selected humeral head (red circle) and the resulting virtual screw trajectories (green lines). All screw lengths are shown on the left side of the graphical user interface. Left image shows screw length determination after plate prepositioning and right after final screw insertion. Graphical user interface by MeVis BrestCare GmbH.

For proximal humeral plating, a stainless-steel marker comprising three cylindrical holes arranged in a defined pattern and two polymer clamps is clipped on a drill sleeve which is attached to a conventional anatomic locking plate (PHILOS ref: 441.901, DePuy Synthes Inc., Raynham, MA, USA) (Figure 1). The nose of the clip locks into the PHILOS guide block to constrain clip rotation. Two anteroposterior X-ray images of the proximal Humerus (including the marker clip) are taken at an angle to determine the position of the humeral head in space in relation to the implant. The boundaries of the humeral head are identified in both images via semi-automatic segmentation. Hereby, the head is estimated as a perfect sphere and best-fit solution candidates are suggested to the operator for final selection. The software calculates screw trajectories based on the marker position and known implant geometry for both X-ray images. By calculating intersection points of the screw trajectories and sphere, proximal screws are virtually truncated to a given tip–joint distance (TJD). Suggested screw lengths (rounded to commercially available increments) are displayed on the software’s graphical user interface (Figure 2).

### 2.2. In Vitro Study

For performance assessment, the Xin1 system was compared to the conventional free-hand method in 12 intact, non-fractured shoulders from 6 formalin-embalmed, full-body specimens (3 male and 3 female), which were pre-used for medical student courses. The experiment was performed at the Department of Anatomy of the Medical University of Innsbruck, Austria. Left and right shoulders were randomized into Xin1 and conventional groups. All operations were performed by a single operator (DK), who was at the time the resident surgeon with moderate surgical experience. For both groups, a deltopectoral approach was created and a PHILOS plate was prepositioned and fixed with a cortex screw in the elongated combi-hole (F-level, Figure 1). A different plate was used for each operation to account for potential manufacturing tolerances from plates and screws. For all operations, a guide-block (Ref. 03.122.056, DePuy Synthes Inc., Raynham, MA, USA) and a drill sleeve (Ref. 03.122.058, DePuy Synthes Inc., Raynham, MA, USA) were used for predrilling. All pilot holes were intentionally drilled into the joint space to enable the screw tip-to-joint distance (TJD) measurement. A C-arm with an image intensifier (Siemens Arcadis Varic, Siemens Healthcare GmbH, Erlangen, Germany) was used for fluoroscopic imaging.

For the conventional procedure, the plate position and screw lengths were estimated via repeated fluoroscopy and by use of a length probe (Ref. 03.122.052, DePuy Synthes Inc., Raynham, MA, USA) with a target TJD of 5 mm. Eight screws (Ref. X12.1xx, 3.5 Locking Screw, TAN, DePuy Synthes Inc., Raynham, MA, USA) were placed using the screw insertion guide (Ref. 03.122.053, DePuy Synthes Inc., Raynham, MA, USA). Only screw hole 7 on the D-level was left empty. Plate and screw placement was verified via fluoroscopic imaging.

For the Xin1 procedure, a marker clip was additionally attached to the drill sleeve (Figure 1). After prepositioning the plate, a first pair of X-ray images was taken at an angle of ~30° in an approximately anteroposterior direction. A tablet computer with Xin1 software was connected to the C-arm via PACS (Picture Archiving and Communication System). Image pairs were transferred to the tablet computer on request of the operator during the procedure. The humeral head was segmented by the operator in both images of a pair on the touch screen via the semi-automatic segmentation approach. All proximal screw lengths to a target TJD of 5 mm were calculated using the system from the image pair and outputted on the screen. Eight proximal screws were placed according to the system suggestion using the screw insertion guide. After screw insertion, another image pair was taken for verification of the implant and screw positions.

For the TJD accuracy measurement, the humeral head was dislocated from the joint, and the screw tip-to-joint distances were measured including the cartilage layer for all screws by inserting a K-wire into the over-drilled hole until touching the screw tip. The insertion depth was then marked with forceps and measured with a caliper (Figure 1). Cartilage thickness was approximated to 1.5 mm by taking repeated samples from the specimens, measuring the cartilage layer with a caliper and then averaging the results. Cartilage thickness was subtracted from the TJD measurements. The absolute TJD error defined as $\left\|\mathrm{TJD}-{\mathrm{TJD}}_{target}\right\|$ was calculated. The procedure time, number of taken X-rays and radiation time were recorded for (1) the plate positioning phase (from procedure start until accepted plate position), (2) screw placement phase (from accepted plate position until last proximal screw placed) and (3) verification phase (from last proximal screw placed until accepted implantation).

Non-parametric independent sample tests (Mann–Whitney U) were performed to compare both techniques regarding accuracy (TJD and TJD error), procedure time, number of fluoroscopic images and radiation time. All analyses were performed using SPSS software version 27 (IBM Corporation, Armonk, NY, USA). For all tests level of significance was set to α = 0.05.

### 2.3. Clinical Handling Test

Eleven patients undergoing plate fixation osteosynthesis with PHILOS were enrolled in a focused registry at a single medical center (UZ Leuven, Leuven, Belgium). The clinical trial was approved by the local ethical committee and the Federal Agency for Medicines and Health Products Belgium (FAMHP, AFMPS/SE/80M0661) and was registered at clinicaltrials.gov (NCT03427112). Informed consent was obtained from all participants before surgery. The standard surgical protocol was maintained, except for attaching an Xin1 marker clip after plate prepositioning. The marker clip was manufactured from medical-grade stainless steel and PEEK (Polyetheretherketon) and was designed for reuse in a standard hospital surgical instruments workflow including autoclaving and cleaning. Intraoperative X-rays from two stages, before and after screw placement, were stored in a DICOM (Digital Imaging and Communications in Medicine) format and were retrospectively evaluated with the Xin1 software. No system feedback was provided to the surgeon during operation. Placed screw lengths during surgery were noted for all operations.

**Figure 3:**
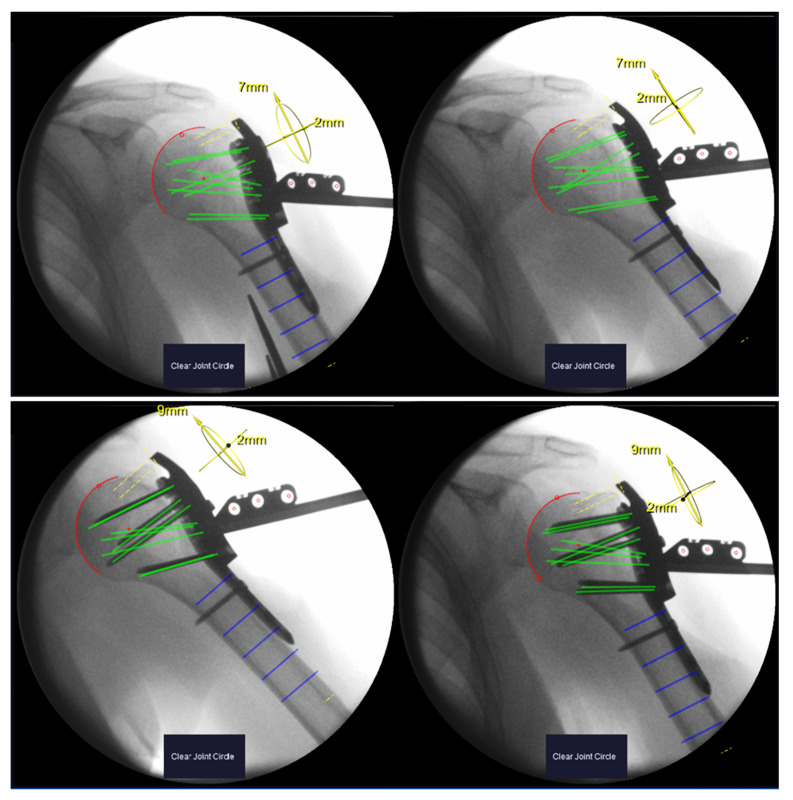
Xin1 software output for an exemplary patient. Top row: image pair before screw insertion. Calculated screw trajectories depicted in green. Segmented humeral head in red. Bottom row: image pair after screw insertion (verification images). Green trajectories coincide with inserted screws.

Image pairs from both stages were formed from the available X-rays and processed with the Xin1 software. An image pair was found valid if the view angle between the images exceeded 15°. So that several valid pairs could be formed, the pair with the largest view angle was selected. Screw lengths and plate positions were computed from the selected pairs for both stages. The plate position is calculated in relation to the anatomy, defined as the offset of the calculated center of the humeral head from the central screw trajectory (screw 7, D-Level) in cranial–caudal and dorsal–ventral directions (Figure 3). 

To investigate the effect of potential head displacement during screw insertion in a real fracture situation, the differences in screw length before and after fixing the plate with screws ($\left\|L_{after}-L_{before}\right\|$) as well as the differences in the plate position were calculated.

Statistically, correlation analysis was performed on the computed screw lengths before screw insertion and the actually placed screw lengths by pooling the patients and calculating Pearson’s correlation coefficient R. 

## 3. Results

### 3.1. In Vitro Study

**Figure 4:**
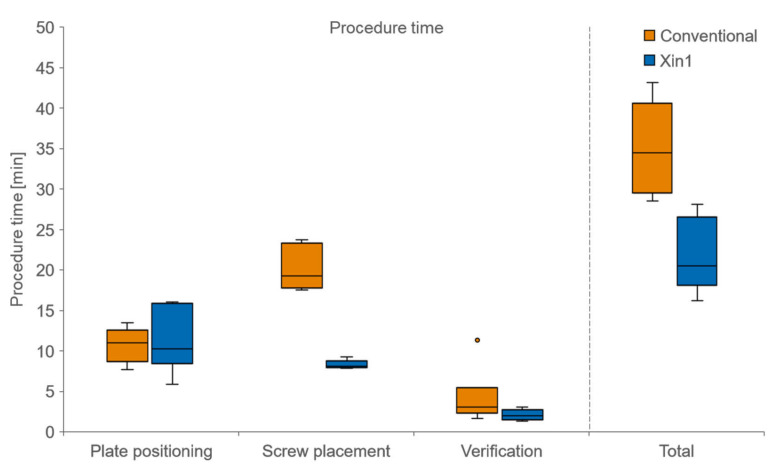
Procedure time for the conventional method versus Xin1 for three phases during the operation and in total time.

The mean procedure time for the conventional technique was 35:03 ± 6:11 min (mean ± SD), compared to 21:41 ± 4:31 min for the Xin1 technique. This total time reduction of 38% when using Xin1 was statistically significant (*p* = 0.002). The time reduction compared to the conventional technique was largest during a screw placement with 59% (Figure 4).

**Figure 5:**
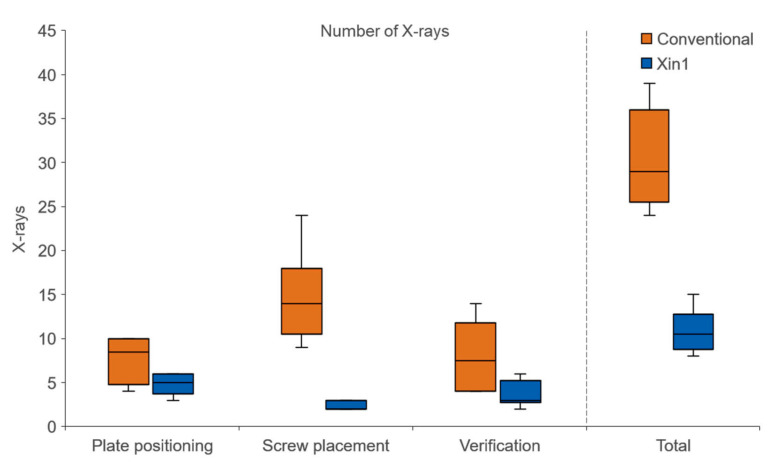
Number of X-ray images taken for conventional and Xin1 groups for three phases during the operation and in total.

The number of fluoroscopic images was on average 30.3 ± 5.7 (mean ± SD) with the conventional technique and 10.8 ± 2.5 images with Xin1. This reduction of 64% when using the Xin1 system was statistically significant (*p* = 0.002). The largest X-ray reduction was found during the screw placement phase with 84% (Figure 5). Accordingly, the mean radiation time for the conventional technique was 23.7 ± 4.1 s, compared to 7.5 ± 2.0 s for Xin1 (*p* = 0.002).

**Figure 6:**
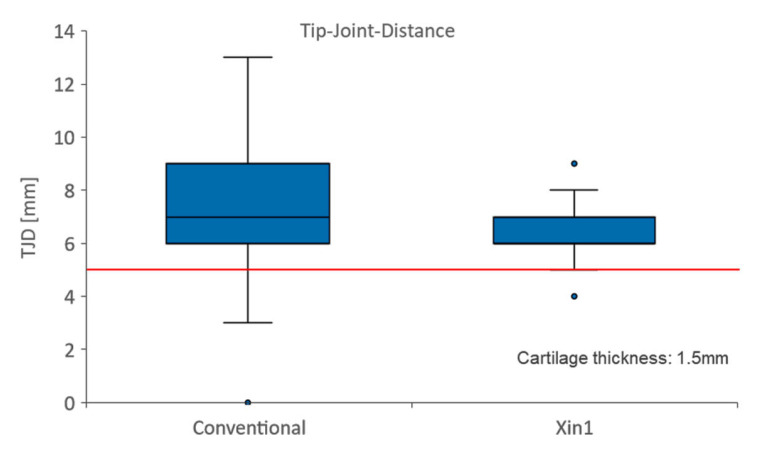
Precision of achieved tip–joint distance (TJD). Target distance was set to 5 mm in Xin1 (red line). In total, 48 screws were placed in 6 human specimens.

In each study group 48 screws were placed. Two joint perforations occurred in the conventional groups, while in the Xin1 group no perforations were observed. The mean tip–joint distance (TJD) was 5.8 ± 2.6 mm (mean ± SD) with the conventional technique, and 5.1 ± 1.3 mm when using Xin1 (Figure 6). This difference was not statistically significant (*p* = 0.053). However, the absolute error of the screw length deviating from the target TJD of 5 mm was on average 2.2 ± 1.6 mm for the conventional technique and 1.0 ± 0.7 mm with Xin1. The error, hence, diminished by 52% when using the Xin1 system, which was statistically significant (*p* < 0.001). 

### 3.2. Clinical Handling Test

Eleven proximal humerus fractures (eight left, three right shoulders) from eight female and three male patients were operated by five different surgeons. The average age of patients at surgery was 67 years and ranged between 47 and 84 years. Fracture types (AO classification) were: 1× 11A2, 1× 11A3, 3× 11B1, 1× 11B2, 4× 11C1 and 1× 11C3. With one exception, where a Philips Zenition 70 system (Koninklijke Philips N.V., Amsterdam, The Netherlands) was used, all intraoperative images were acquired with a Siemens Arcadis Varic (Siemens Healthcare GmbH, Erlangen, Germany) C-arm. 

Handling of the marker clip in the hospital environment and during surgery was feasible in all patients and did not interfere with hospital processes or surgical procedures. No adverse events concerning the use of the marker clip were recorded for the 11 performed operations. 

For the retrospective image analysis, five out of eleven patients needed to be excluded for various reasons: for example, one patient was excluded due to the intraoperative use of a wrong drill sleeve, leading to the mispositioning of the marker clip. For two patients, no images after screw insertion were stored to the C-arm. For another two patients, the image pair view angle was too narrow for calculation.

Xray images of the remaining six patients underwent retrospective evaluation. Robust marker detection, humeral head segmentation and screw length determination were achieved in all image pairs. The view angle between the images of a pair was on average 27° (range 19°–34°). The calculated screw lengths before and after screw placement differed on average by 1.2 ± 1.7 mm. In five out of six patients, this difference was ≤2 mm for all screws. In one patient (#10), differences up to 8 mm were found. 

**Figure 7:**
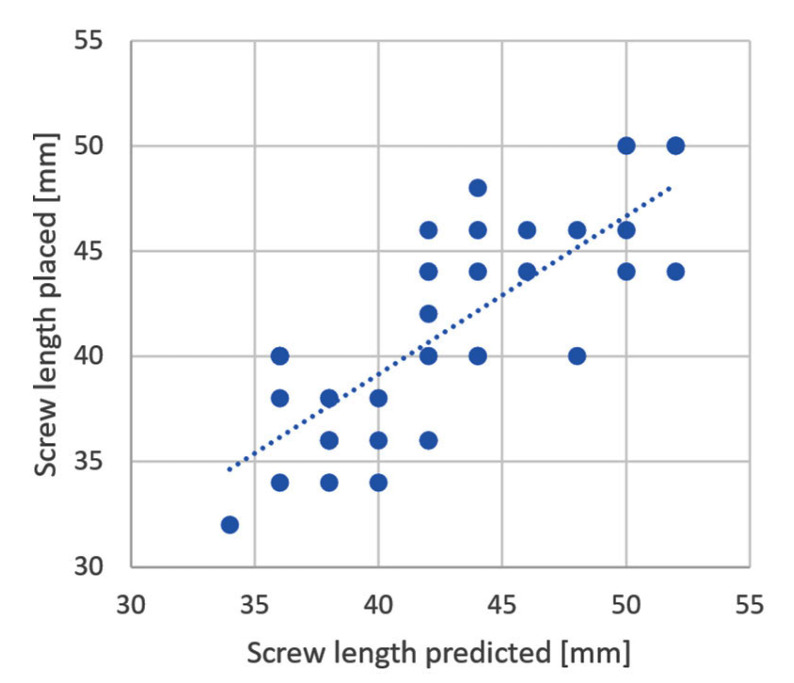
Correlation between Xin1 computed screw lengths (before screw placement) and actually placed screw lengths (no system output provided to the surgeon).

A total of thirty-seven screws were placed in the six evaluated patients. Used screw lengths ranged from 32 to 50 mm. The Xin1 calculation before screw placement correlated significantly with the actual screw lengths implanted (R = 0.78, *p* < 0.001, Figure 7). The maximum deviation between the Xin1 output and placed screw length was 8 mm.

Maximum plate offset before screw placement was 5 mm in a dorsal–ventral direction and 9 mm in a cranial–caudal direction in respect to the center of the humeral head. After placing the screws, the relative plate position shifted at a maximum of 4 mm for five out of six patients. In one patient (#10), the plate was displaced by 10 mm in the dorsal–ventral direction after screw placement according to the Xin1 measurement (Table 1).

## 4. Discussion

The Xin1 implant positioning system, as introduced in [5], uses existing imaging modalities such as a C-arm to guide the surgeon in the 3D anatomical environment, where otherwise experience and strong spatial perception are required. The system was designed with simplistic thinking in mind to augment established workflows and overcome the common shortcomings of surgical navigation systems, such as complexity, setup and takedown efforts as well as high costs [7]. The Xin1 principle was abstracted and developed into various prototype application modules, as summarized in [5]. In vitro performance of a first module for distal nail interlocking was described in [6]. The performance and clinical application of another module for controlling rotational osteotomies was outlined in [8], whereas this work focused on a prototype module for proximal humerus plating. 

Anatomical locking plates such as the PHILOS system stabilize proximal humerus fractures by spanning a multitude of screws in the often-osteoporotic head segment. It is aimed to anchor the screw tips in the subchondral bone region, close to the cartilage layer. The task is surgically demanding due to an eminent risk of screw penetration into the joint space while trying to judge an oblique screw path inside a spherical anatomical structure from planar X-ray projections. On the other hand, too-short screws miss anchorage [3]. Failures and complications of plated osteoporotic proximal humeral constructs are frequent problems [9]. Complication rates are reported between 28% [10] and 79% [2]. A significant portion of these cases can be attributed to implant and screw misplacement [4]. An increasing number of older patients with compromised bone mass will amplify the problem in the future and demand increased care and precision in surgical execution. The Xin1 development was motivated by these current and future clinical needs with the rising incidence of osteoporotic fractures.

In the scope of this work, the prototype Xin1 system was evaluated in terms of performance in both postmortem and clinical settings. The system estimates the plate position and calculates all required screw lengths to reach into the subchondral bone area from a pair of conventional C-arm images. Xin1 outperformed the conventional freehand technique in terms of surgical precision (52% error reduction), procedure time (38% shorter) and radiation exposure of OR personnel and the patient (64% less X-rays). Particularly for medical professionals, the continuous exposure to ionizing radiation during surgery is a frequently addressed problem [11,12,13]. The increased effectiveness of surgical procedures and consequent time reduction gain focus in an increasingly economically driven health care environment. 

The postmortem experiment was performed on full-body specimens with compromised soft tissue mantle due to embalmment and previous use. However, we believe that the experiment carried high relevance for judging the technology before entering into clinical testing. One methodological issue, though, could not be neglected, which was the absence of bone fractures. 

**Figure 8:**
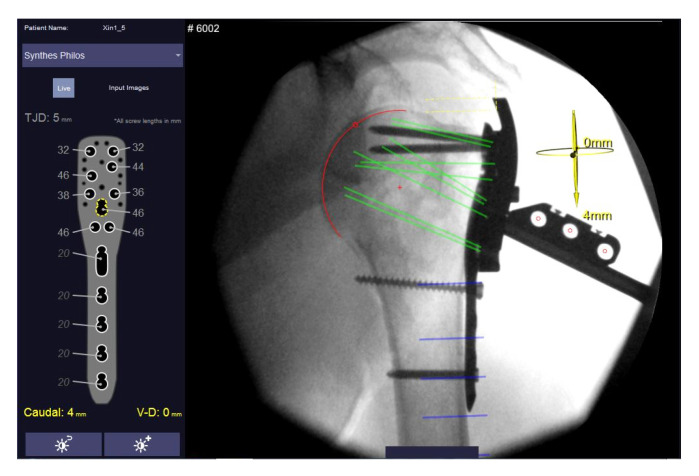
Intraoperative X-ray of one case with tilted screws (PHILOS A-level) as apparent by non-alignment with the calculated screw trajectories (green lines).

In a second phase, we hence conducted a clinical registry on fracture patients to investigate the potential effect of bone fragment shifts on the system output, and at the same time to analyze the clinical handling of the Xin1 marker clip. Safe handling was shown in all 11 included patients by the absence of device-related adverse events. To minimize risks to the patient at the current state of system development by potentially falsified system outputs, X-ray image analysis was performed in a retrospective fashion without providing system information to the operator during surgery. The missing feedback in the operating theatre reduced risks but also led to a high number of patients, who needed to be excluded from the image-based evaluation (five out of eleven). These dropouts are deemed to be artifacts from the retrospective study design and may be avoided in the future when real-time feedback is provided to the operator. A stereotactic system always relies on an angular spread of at least two view ports to reconstruct a 3D situation [14]. Without system feedback to the surgeon, the view angle between the two images of a pair was often too narrow and did not allow for accurate calculation. In one patient, a wrong drill sleeve was used, leading to a wrong marker position and consequently a false calculation. Additionally, marker shifts are likely to be detected when system feedback is provided. A clip-on approach has the advantage that already available implants can be utilized but carries the risk of the misplacement of the marker. Integrated holes in dedicated instruments or implants could overcome this issue in the future. Another important aspect which became apparent during the study was the need for the use of a screw guide. While utilizing a drill sleeve for pilot hole drilling, screws may still deviate when inserted in a freehand manner and obliquely lock into fixed angle plate holes (see exemplary image, Figure 8). As known from Lenz et al. [15], tilted screw insertion diminishes the holding strength in the plate, and obviously compromises guiding systems such as Xin1. In any case, the use of a screw guide should be mandatory in locked plating. 

Besides these general observations, the clinical study confirmed the feasibility of the marker clip to be embedded into hospital and surgical workflows. The system software demonstrated robust performance in presence of real-life bone fractures. Certain head fragment shifts due to manipulations during the screw insertion were suggested by differences in the computed screw lengths and measured plate position before and after screw insertion. Shifts were generally small, but in one case they reached 10 mm in the dorsal–ventral direction. Clinical consequences such as screw perforation into the joint cannot be excluded in such cases. It is therefore proposed to perform a verification measurement with the Xin1 system after the head fragment has been fixed in place with the first screw. On a critical note, it cannot be ruled out that the measured differences are artifacts related to inaccuracies of the system rather than describing actual bone fragment shifts. In this regard, the segmentation procedure to determine the position of the humeral head, still relying on user input, might play an important role. Inaccurate determination of the humeral head boundaries will impact the accuracy of the system. Fully automated segmentation may be desired in the future. In a previous bench experiment [5], the angular precision of the Xin1 system was measured to be <1° and the precision of the screw length determination was 1.2 mm (both standard deviation) under defined conditions. However, rigorous performance validation must follow before introducing the Xin1 system into clinical practice. Another reason for fracture shifts or inaccurate calculation can be the operator’s technique for acquiring the angulated X-rays views. It is common clinical practice to rotate the patient’s arm when adjusting the X-ray projection. This, however, can lead to a relative displacement of bone fragments. It is therefore preferred to pivot the C-arm instead of moving the shoulder. Despite these methodological issues, the calculated screw lengths before screw insertion correlated with the actual screws placed by the surgeon who was blinded to the Xin1 output. This observation underlines the general feasibility of the system and suggests a strong potential of such assistance devices to significantly simplify orthopedic workflows and improve surgical precision. 

Study limitations are summarized as the following: The postmortem experiment was performed on unfractured embalmed body donors with compromised soft tissues. The effect of intact soft tissues and fractured proximal humerus was hence difficult to estimate from this sub-study. Furthermore, the tip–joint distance evaluation assumed a constant cartilage layer thickness, which naturally varies between locations and individuals. Procedure time assessment did not take system setup and takedown durations into account, and thus represents only a net value. The clinical registry allows only for preliminary conclusions at this early development stage because of the missing system feedback in the operating room. This also led to a considerable number of dropouts lowering the sample size for image evaluation. As desired for a clinical handling investigation, several surgeons shared the surgeries. This, on the one hand, gave valuable insights about marker clip usage in different hands, but might have contributed to the large number of excluded datapoints for image analyses. However, several confounders regarding the Xin1 approach for proximal humerus plating could be identified from the studies, including: potential marker movements, risk for use of wrong implants and instruments, omitted use of a screw guide, bone fragment shifts due to surgical manipulation, a too-narrow X-ray view angle or the segmentation procedure of the humeral head requiring manual interaction. Furthermore, some technical limitations rendering validation and regulation of such assistance devices difficult are to be mentioned. Optical image distortion of older image intensifiers can drastically influence the system accuracy. Flat panel-based C-arms solve this issue and currently replace image intensifiers around the globe. Still, interfacing to the imaging modalities remains an issue. A variety of analog and digital output protocols make connecting external devices difficult. Important parameters such as flip or rotation flags are not transmitted. The ability to run third party image processing software directly on the C-arm would be an important prospect for future developments, dramatically increasing usability and effectiveness of assistance technologies such as Xin1.

## 5. Conclusions

This study demonstrated the potential of a newly proposed assistance technology for implant positioning in proximal humerus plating using conventional X-ray projections. In an in vitro setting, the system significantly improved surgical precision, reduced X-ray exposure and shortened surgery time. In a clinical handling test, the device demonstrated seamless embedding into existing clinical workflows and robust performance in a real-life fracture situation. However, certain issues crystallized and need to be addressed before placing such system into clinical practice. A strong potential to improve patient outcomes in the rising incidence of osteoporotic humeral fractures is eminent.

## Figures and Tables

**Table 1:** Patient details, view angle of image pairs and plate position before (pre) and after (post) screw placement for the 6 patients undergoing retrospective image evaluation. D–V: dorsal–ventral plate position; negative sign: head center is located ventral from plate. C-C: cranial-caudal plate position; negative sign: head center is located caudal from plate. f: female, m: male; L: left, R: right.

Patient #	Gender	Side	Fracture	Pre View Angle [deg]	Post View Angle [deg]	Pre D-V [mm]	Post D-V [mm]	Diff [mm]	Pre C-C [mm]	Post C-C [mm]	Diff [mm]
2	f	L	11A3	31	25	−1	−2	1	5	4	1
3	f	R	11C1	31	30	−5	−5	0	8	9	1
4	f	L	11C1	24	22	−1	−1	0	7	8	1
6	f	L	11C1	27	31	4	0	4	4	3	1
10	m	L	11A2	34	27	−1	9	10	8	8	0
11	m	R	11B2	24	19	−1	−3	2	0	2	2

## Data Availability

Not applicable.
